# Spatiotemporal determination of photoinduced strain in a Weyl semimetal

**DOI:** 10.1063/4.0000263

**Published:** 2024-09-30

**Authors:** Jianyu Wu, Amit Kumar Prasad, Alexander Balatsky, Jonas Weissenrieder

**Affiliations:** 1 Light and Matter Physics, School of Engineering Sciences, KTH Royal Institute of Technology, SE-100 44 Stockholm, Sweden; 2 Nordita, Stockholm, Sweden; 3 Department of Physics, University of Connecticut, Storrs, Connecticut, CT 06269-3046, USA

## Abstract

The application of dynamic strain holds the potential to manipulate topological invariants in topological quantum materials. This study investigates dynamic structural deformation and strain modulation in the Weyl semimetal WTe_2_, focusing on the microscopic regions with static strain defects. The interplay of static strain fields, at local line defects, with dynamic strain induced from photo-excited coherent acoustic phonons results in the formation of local standing waves at the defect sites. The dynamic structural distortion is precisely determined utilizing ultrafast electron microscopy with nanometer spatial and gigahertz temporal resolutions. Numerical simulations are employed to interpret the experimental results and explain the mechanism for how the local strain fields are transiently modulated through light–matter interaction. This research provides the experimental foundation for investigating predicted phenomena such as the mixed axial-torsional anomaly, acoustogalvanic effect, and axial magnetoelectric effects in Weyl semimetals, and paves the road to manipulate quantum invariants through transient strain fields in quantum materials.

## INTRODUCTION

Topological quantum materials represent a class of solid-state systems distinguished by their resilient interface states and quantized bulk response coefficients, stemming from the intricate nature of their electronic wavefunctions.^1^ Their distinctive traits position them as promising platforms for storing and manipulating quantum information.^2,3^ The manipulation of topological invariants in these materials may lead to exotic states and its extension to novel applications.^4^ Lattice strain provides a natural means of tuning the topological invariants since strain directly modifies the electron–lattice interaction and can change the underlying crystalline symmetry on which the topological properties depend.^5,6^ However, modulation of the local strain field has in most studies been restricted to the application of static pressure under equilibrium conditions,^6–8^ which pose challenges for the understanding and manipulation of transient topological states. Light–matter interaction in two-dimensional (2D) and topological materials provides an avenue for inducing emergent, non-equilibrium properties and for controlling the function on ultrafast timescales.^9,10^ Illumination by light leads to excitation of electrons that is rapidly followed by an energy transfer to the lattice, through electron–phonon scattering. When using ultra-short light pulses, this process can induce coherent atomic displacements.^11^ The role of such phonons in dynamical processes has been the subject of several time-domain studies, for example, photoinduced phase transitions of charge-density wave materials and the modification of magnetism.^12–15^ Moreover, the application of topological concepts to explore phonon-related properties has catalyzed the emergence of the fields of topological and nonlinear phononics.^16–22^ Therefore, understanding phonon dynamics and its relation to material properties stands as a pivotal factor in advancing functionality and applications within the realm of topological materials.

Weyl semimetals are materials where valence and conduction bands cross in single points, the Weyl nodes. The presence of Weyl nodes can be attributed to the material's symmetry, implying that a material's topological properties can be manipulated through atomic-scale lattice distortions.^23–25^ The type II Weyl semimetal WTe_2_ is a prominent example, where ultrafast light excitation induces a transition from the topological *Td*-WTe_2_ phase to the trivial 1T^*^ phase.^23^ The phase transition proceeds under the effect of shear strain induced from photodoping and the excitation of interlayer A^1^ optical phonons.^26^

Any lattice deformation, e.g., lattice mismatch, local bending, and defects, will induce a local strain field.^27–29^ The combination of static fields with strain modulation from dynamic processes offers opportunities to manipulate both the local atomic structure and the electronic properties. For example, the transition pathway in *Td*-WTe_2_ can be selectively tuned to a desired product phase (either orthorhombic 1T^*^ or monoclinic 1*T*′) through the application of a static strain field derived from local sample morphology.^30^ Additionally, theory suggests that Weyl semimetals exhibit a mixed axial-torsional anomaly and an axial magnetoelectric effect in the presence of structured strain and axial torsion.^31,32^ To activate these anomalies, realistic experiments with spatially varying strain distribution and a transient displacement vector of phonons through the Weyl semimetal are required. Such experimental conditions necessitate the observation and manipulation of transient structural modulation as well as strain distribution at the microscopic level.

This study explores transient structural deformation and strain modulation in WTe_2_, with a special emphasis on the microscopic regions exhibiting strain defects. The combination of static strain fields at local line defects and dynamic strain, driven by photo-induced coherent acoustic phonons, leads to the formation of local standing waves at the line defects. The dynamic structural distortion is precisely determined with ultrafast electron microscopy (UEM), at nanometer-scale spatial precision and gigahertz-scale temporal resolution. To interpret the experimental findings, we employ numerical simulations of the structural dynamics. This research sets the experimental foundation for exploring the predicted mixed axial-torsional anomaly, the acoustogalvanic effect, and axial magnetoelectric effects in Weyl semimetals.^31–33^

## RESULTS AND DISCUSSION

**FIG. 1.**
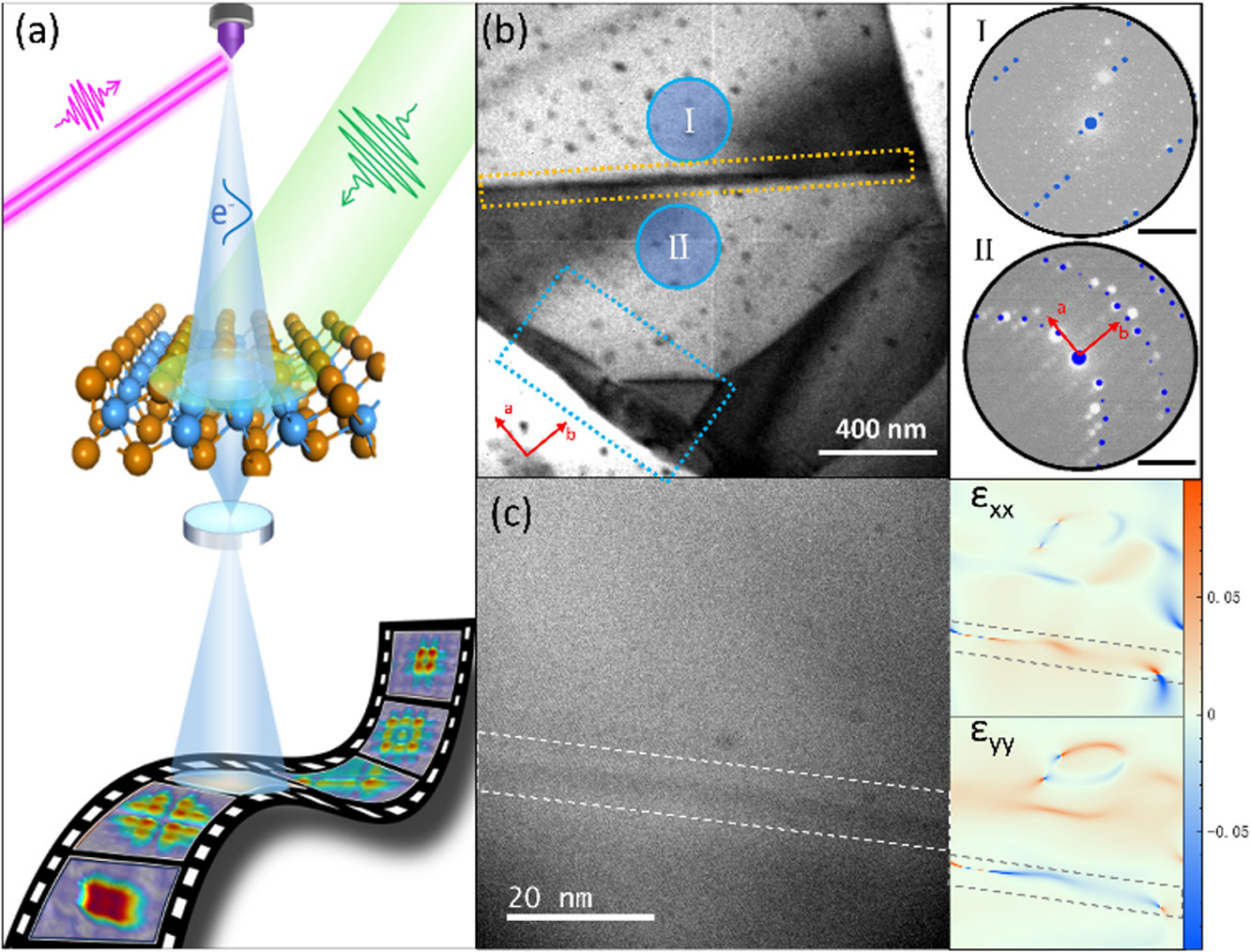
(a) Schematic illustration of the principle for the time-resolved electron microscopy analysis, including the WTe_2_ sample. The purple beam represents the 258 nm femtosecond laser beam illuminating the photocathode to generate the pulsed electron beam (blue, probe). The green beam represents the 515 nm laser that excites the sample (pump). (b) Bright-field TEM image of the WTe_2_ sample before photoexcitation (time zero) (supplementary material video). The yellow dashed rectangle indicates a local curvature of the sample, the blue dashed rectangle indicates a section of sample magnified in Fig. 2, and the blue circles I and II are positions for the selected area electron diffraction patterns (SAED, I and II). The crystallographic directions (a) and (b) are indicated by the red arrows. Scale bar: 10 1/nm. (c) High-resolution TEM image of the line defect (bend) in (b). The curvature is indicated by a white dashed rectangle. The right panel insets show strain distributions in the horizontal direction (ε_xx_) and vertical direction (ε_yy_) calculated from the TEM image in the left panel.

The operating principle of the UEM instrument is illustrated in Fig. 1(a). A WTe_2_ sample is excited by a 515 nm laser pulse and then probed by photoelectrons. Additional experimental information can be found in the “Method” section. A bright field image of a single-crystal WTe_2_ sample is shown in Fig. 1(b). The sample has a peninsular shape with its upper left corner connected to a larger flake (Fig. S1, supplementary material).^50^ Several line defects are observed, one of the line defects is indicated by a dashed yellow rectangle in Fig. 1(b). The sample orientation on either side of the line defect resides in distinct zone axes. The selected-area electron diffraction (SAED) patterns in insets I and II of Fig. 1(b) illustrate the different local orientations on either side of the line defect. The SAED pattern from zone I exhibits a 2.7° tilt from the principal [001] crystallographic zone axis, away from the incident electron wave vector and toward the a-axis of the sample. In zone II, the local sample orientation exhibits a tilt of 1.4° along the a-axis and 3° along the b-axis, away from the incident electron wave vector. The overlayed blue spots in the insets represent simulated diffraction patterns.^50^ The different local orientations of the sample result in a bent region at the junction. The bright-field image of the bending region exhibits dark contrast because of local changes in the Bragg conditions. These intensity contours are termed equal inclination fringes or bend contours.^34^ One bend contour with a 5 nm width is shown in the high-resolution transmission electron microscopy (HRTEM) image in Fig. 1(c). Line defects are commonly accompanied by strain fields.^30^ Through geometric phase analysis of the HRTEM results, the strain distribution can be visualized in strain maps.^35^ The strain maps generated from the region of the line defect in Fig. 1(c) show an inhomogeneous strain distribution in both the horizontal (ε_xx_) and vertical (ε_yy_) directions of the image. The bend line defects (wrinkle defects) are formed during the sample preparation process that includes transfer to the transmission electron microscopy (TEM) grid. The small black spots, scattered across the sample and supporting membrane, are contaminants from the sample preparation procedure.

**FIG. 2.**
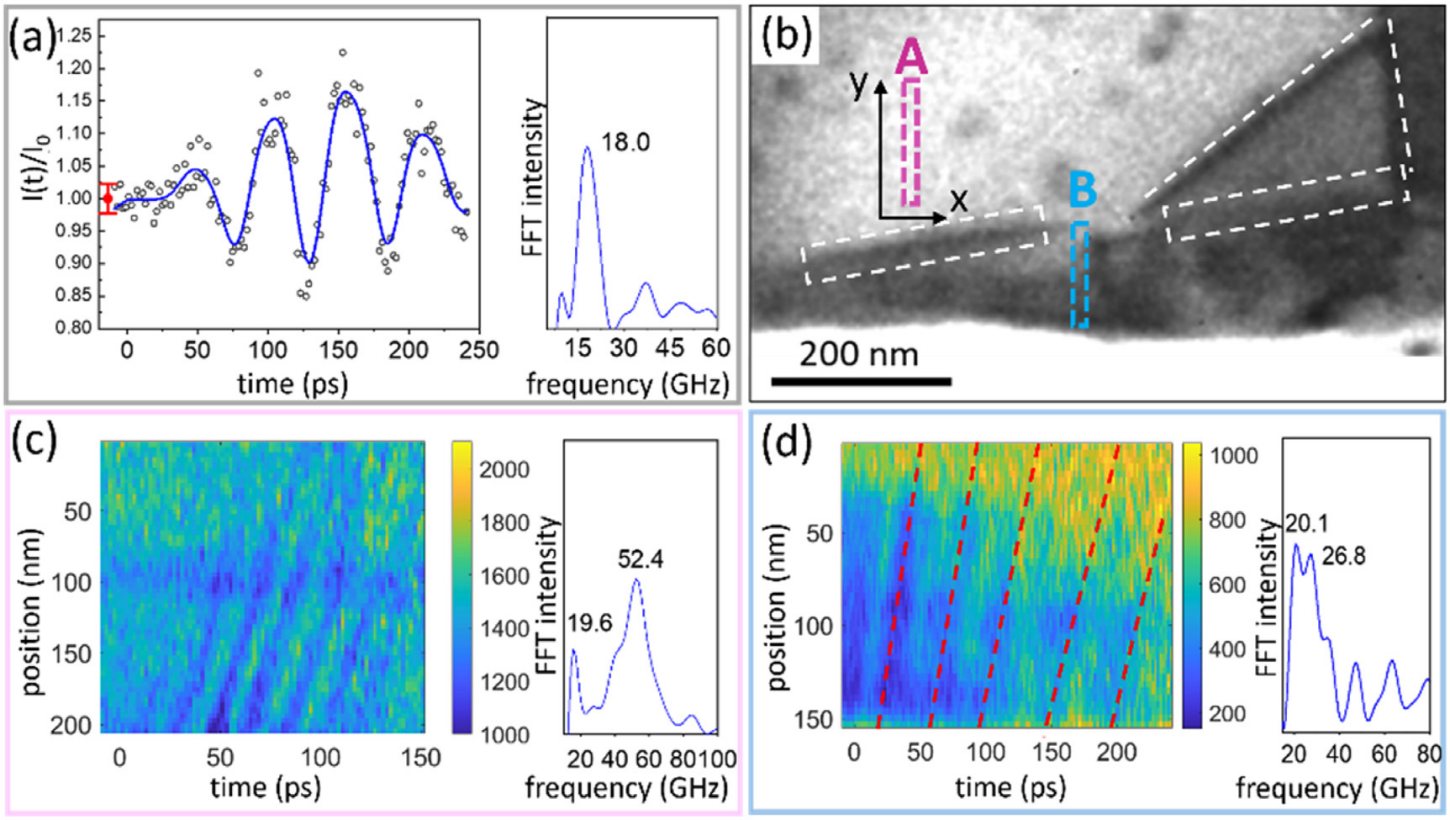
Local dynamics of acoustic phonons. (a) Temporal variation in the relative image intensity integrated from zone II and FFT spectrum of the time trace. The black circles in the left panel represent experimental data, and the blue line is a guide for the eye. The error bar (red) is extracted from the data before time zero with whiskers showing a confidence level of 95%. (b) Magnified TEM image of sample edge and bend contours, the white dashed lines and rectangles indicate the positions of bend defects, and rectangles A (pink) and B (blue) indicate the positions of ROI A and ROI B, respectively. (c) and (d) STCP generated from ROI A (c) and ROI B (d) and FFT of the changes in intensity. Note the different time scales in (c) and (d). The red dashed lines serve as a guide for the eye for the wavefronts.

Upon laser excitation at 515 nm, coherent acoustic phonons are generated and propagated across the sample in the plane of the sample as well as in the thickness direction of the sample (c-axis). The propagation of acoustic phonons within the sample leads to small angular perturbations of the lattice, resulting in deviations of a few milliradians in the local Bragg condition. As a result, dynamic contrast is observed in the local morphology in the bright field TEM images.^36^ In zone II of Fig. 1(b), a spatially stationary oscillation is observed with a frequency of 18.0 GHz. The normalized pixel intensity of the TEM image time series is shown in Fig. 2(a). Changes in pixel intensity are observed after time zero (time of arrival of the laser pulse at the sample) as oscillations around its value before time zero. Such dynamic response is commonly observed from phonon modes confined by the terminating surfaces of the sample.^37,38^ The frequency of the oscillation is determined by the speed of sound in the c-direction of the crystal and the local thickness of the specimen. With a local sample thickness of 51 nm, as measured by electron energy loss spectroscopy (EELS) (Fig. S4, supplementary material),^50^ the acoustic wave propagation velocity along the c-axis is calculated to 1.82 nm/ps, close to the wave velocity calculated from the elastic constant (Table S2, supplementary material).^50^ We can, therefore, assign the coherent oscillation in zone II as a longitudinal wave along the principal crystallographic c-axis direction. We observe a growing amplitude in the initial oscillations, as has been reported in a prior study on a sample containing defects.^37^ The increase in amplitude can be attributed to irregularities in the sample structure, including defects and local tilt.

The time-resolved results do show not only spatially stationary periodic oscillations but also wavefronts propagating across the sample. The traveling waves can be observed propagating from the edges toward the center of the sample in Video 1 (supplementary material video). Coherent acoustic waves are often found to start at the edge of samples, mainly because of local changes in light absorption, boundary condition effects on stress, and the discontinuity in acoustic impedance at the edge.^39,40^ The acoustic wave propagates along the y-axis in Fig. 2(b). We define two rectangular regions of interest (ROI) with widths (x direction) of 4 nm and long sides in the y direction. ROI A is at a flat region of the sample above the bend contour, while ROI B is at an area exhibiting irregular morphology in proximity to the sample edge. The pixels in the ROI's width direction are integrated to form a one-pixel column along the y spatial direction. The columns at each time delay are combined with a space-time contour plot (STCP).^41^ The STCPs from ROI A and ROI B are shown in Figs. 2(c) and 2(d). The wavefronts from the propagating waves within each ROI appear as dark bands. The wave velocities of the wavefronts can be extracted from the slope of the bands. The wavefronts in Fig. 2(c) maintain a consistent slope and period, indicating a non-dispersive nature of the propagating waves over the 150 ps following excitation. The velocity is determined to be 4.06 ± 0.45 nm/ps (where the error represents the standard deviation of the velocities extracted from the first five detected wavefronts in the STCPs). This velocity agrees well with the calculated longitudinal wave velocity in WTe_2_ along the b-axis (4.19 nm/ps). The large difference in wave propagation velocity along the c- and b-axes is supported by the first-principle calculations and is ascribed to the weak inter-layer vdW interactions along the [001] direction.^42^ The frequency of the in-plane propagating wave in ROI A is 52.3 GHz. Note that a second peak is observed at the frequency of 19.6 GHz, corresponding to the acoustic phonon propagating along the c-axis as discussed previously. It is worth noting that the frequency of the c-axis phonon exhibits a slight shift between zone II and the current ROI. The reason for this shift can be traced to a variation in the sample thickness. The local thickness of the sample at ROI A is 42 nm, slightly thinner than the 51 nm at zone II. The presence of this peak points to the importance of determining the local (microscopic) frequency as even a slight difference in thickness will result in broadening of the global frequency, especially for samples where atomically smooth interfaces are difficult to produce.

The wave propagation in ROI B exhibits a distinctive dispersive behavior, as depicted in the STCP shown in Fig. 2(d). The slope of the wavefronts (indicated by red dashed lines as a guide for the eye) gradually decreases from the first to the fifth wavefront, indicating a dispersion in wave velocity. The value of the velocity extracted from the STCP decreases from 5.0 to 2.5 nm/ps during the first five periods (approximately 250 ps). The velocity dispersion is accompanied by a red shift of the wave frequency, which is reflected in a broadening of the peak extracted from FFT such that it extends from 26.8 to 20.1 GHz. Such dispersion feature is consistent with previous studies of Lamb waves in germanium wedges and LaFeAsO lamellae, in which both the samples show an irregular morphology and varying thickness.^43,44^ When Lamb waves propagate in plate-like structures, they are confined and reflected from the surfaces of the structure. The guided wave can be modulated by changing surface boundary conditions and thus introduces the observed dispersion.^45^ The irregular sample morphology at ROI B thus contributes to the local dispersion of the wave.

**FIG. 3.**
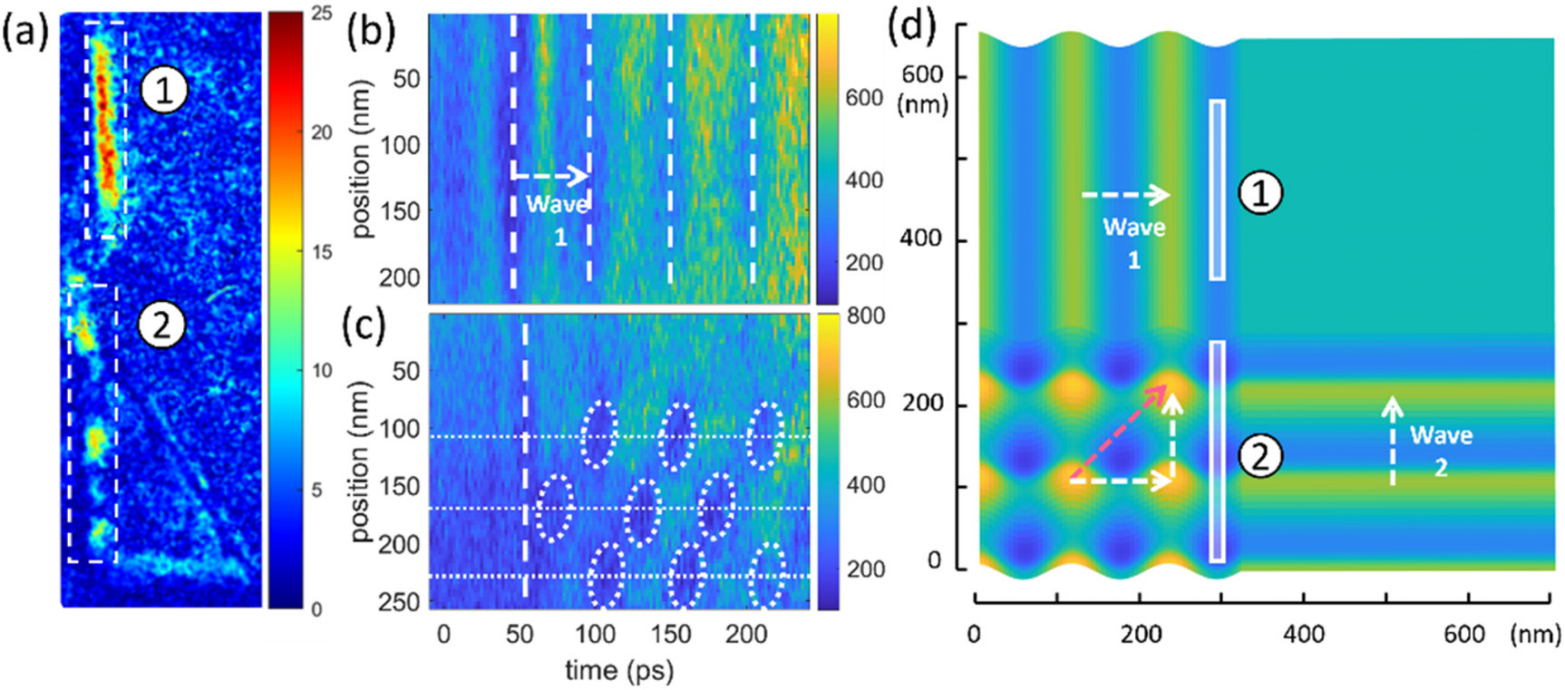
Dynamics of bend contours. (a) Spatial frequency map at 20.0 GHz of a region with two bend contours, with the false color scale representing the FFT amplitude. The image is rotated 90° clockwise from Fig. 2(b), and the white dashed rectangles labeled 1 and 2 represent the position of defects indicated by the white dashed rectangles in Fig. 2(b). (b) and (c) STCP generated from the line profiles of rectangles 1 (b) and 2 (c). The vertical dashed lines and ellipses indicate wavefronts at maximum amplitude. The horizontal dashed lines serve as guides to the eye for the oscillations. (d) Numerical simulation of the interference of two propagating waves. Rectangles labeled 1 and 2 represent defect 1 and defect 2, respectively. Defect 1 is excited only by one wave (wave 1), while defect 2 is excited by the superposition of two waves (wave 1 and wave 2).

A series of TEM bright field images are used to analyze the dynamics of a region containing line defects under the influence of coherent acoustic phonons. Spatial positions for strong oscillations are identified using spatial frequency mapping in Fig. 3(a). A 20 GHz oscillation is observed at positions of the bend contours indicated by 1 and 2 [Fig. 3(a)]. Interestingly, the two different line defects display distinct behaviors corresponding to their local morphology. At line defect 1, a strong 20 GHz oscillation is observed across the entire length of the line defect, whereas line defect 2 exhibits a spatial distribution in oscillation amplitude. Line defect 2 shows three vibrational hotspots exhibiting strong oscillations, with the regions in-between hotspots remaining essentially static. Line profiles with a width of 7 nm are extracted along the line defects to generate STCPs [Figs. 3(b) and 3(c)]. The line defects undergo periodic in-plane oscillatory motions, alternating back and forth, as acoustic waves traverse the region. This results in bright and dark contrast in the STCPs. We interpret the first dark band in the STCP as the arrival time of the acoustic wave. In Fig. 3(b), we observe the first band at 45 ps. This is the expected time of arrival of an acoustic wave propagating from the sample edge [shown in Fig. 2(b)] to this line defect. The oscillation of line defect 2 begins 4 ps after line defect 1 (as indicated by the white dashed lines). This phase shift can be rationalized by the slightly longer distance between the sample edge and line defect 2 (127 nm) compared to the distance between the sample edge and line defect 1 (110 nm). The estimated time delay for the wave propagation is 3.4 ps, using the previously established wave velocity of 5.0 nm/ps. Given that we observe the same oscillation frequency (20 GHz) at the defects (1 and 2), we can conclude that the observed oscillations for these defects are driven by propagating wavefronts (wave 1) originating from the sample edge. After the first wavefront, the STCP plot extracted from line defect 2 exhibits a spatially discontinuous oscillation pattern: strong oscillations appear at the local spatial positions of 110, 170, and 230 nm. Positions in-between remain quasi-static. This indicates that the dynamics of line defect 2 is a result of two interfering wavefronts, with the second wavefront (wave 2) propagating at an angle with respect to the first wavefront. The spatial position for the formation of the second wave can be assigned to features in the local sample morphology: a triangle-shaped defect group consisting of several line defects that can be observed in Fig. 3(a). Since line defect 2 is directly connected with the other line defects, oscillation of any one of these defects will lead to a perturbation of the intersection between the line defects. The in-plane motion of the connected defects will serve as a source for the second wave perturbing line defect 2. Wave 1 propagates orthogonally to the line defect, and the wavefronts propagating along the line defect are assigned to wave 2. The velocity of wave 2 can be extracted from the wavefronts in the STCP as 2.67 ± 0.22 nm/ps. This wave velocity is close to the a-axis shear velocity (transverse wave), as calculated from the elastic constants (2.50 nm/ps). Thus, the STCP is interpreted as an interference pattern from the two wavefronts. The cartoon in Fig. 3(d) illustrates the superposition of two propagating waves at a line defect. Wave 1 propagates perpendicular to the line defect, while wave 2 propagates along the defect. The superposition of the waves leads to a direction of propagation of the resulting wave at an angle to the line defect. The red dashed arrow in Fig. 3(d) indicates the propagation direction of the superpositioned wave. The observation of an interference pattern of the wavefronts implies the feasibility of nanostructural modulation through the superposition of acoustic phonons.

**FIG. 4.**
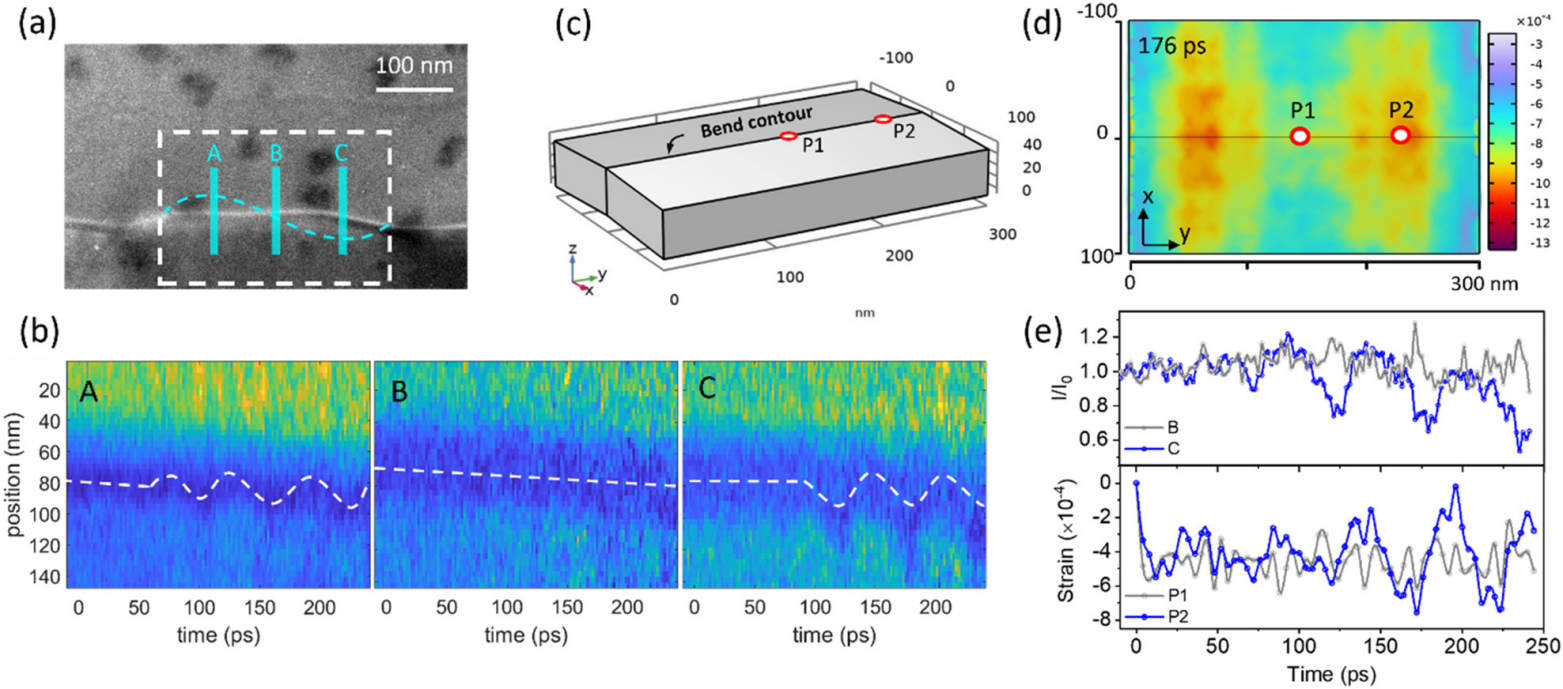
Standing wave in a line defect. (a) Image of sample with a line defect, the dashed blue line indicates the position of the standing wave at the defect, and the blue lines labeled with A–C indicate positions for line profiles used for STCPs in (b). The white dashed rectangle represents the region for simulation. (b) SCTPs generated from line profiles A, B, and C in (a). The white dashed lines serve as guides for the eye. (c) Model used in the COMSOL simulation. The sample is bent 3° from the horizontal to form a bend contour at the center. (d) Snapshot of the simulated strain profile in the x-y plane of the sample at 176 ps. The black line in the center represents the position of the line defect. P1 and P2 indicate two spatial positions for which the time-dependent strain is traced in (e). (e) The upper panel shows the experimental change in pixel intensity of regions B and C. The lower panel shows the simulated time-dependent volumetric strain at positions P1 and P2.

A standing wave is formed from the superposition of two traveling waves propagating in opposite directions. The observations shown in Fig. 4 are in agreement with what is expected from a line defect [located in yellow dashed rectangle in Fig. 1(b)] perturbed by a standing wave. The dynamics of the line defect can be characterized by periodic oscillations (anti-nodes) at specific spatial positions and stationary states (nodes) at other spatial positions. Three line profiles, A–C, are extracted perpendicular to the line defect for the generation of local STCPs. At position B [Fig. 4(b)], the line defect remains static throughout the time delay scan, consistent with a node-like behavior. In contrast, anti-node positions [panels (A) and (C) in Fig. 4(b)] exhibit periodic oscillations of the local curvature. An oscillation phase shift of π is observed between the anti-node positions A and C, as expected from the dynamics of a standing wave. From the analysis, we conclude that the standing wave results in an in-plane periodic modulation of the sample at the line defect, where the discontinuities of the line defect near positions A and C serve as the sources of the waves. Note that local atomic displacements will be considerably less than 10% of nearest-neighbor atom distance (at the picometer level).^46^ However, the in-plane displacement of the line defect curvature observed in Fig. 4 is approximately 20 nm. A previous study has reported a similar oscillation amplitude of line defects.^38^ The relatively large oscillations observed by UEM can be attributed to structural distortions induced by local stress, as the local strain field is subject to modulation by the propagating acoustic waves. The acoustic wavefronts will modulate the local curvature at the line defect. Even subtle changes in curvature will result in significant influence of the local Bragg conditions, giving rise to the observed oscillatory contrast surrounding the line defect. This rationale is further supported by the strain field analysis in the following text.

Based on the findings presented above, we infer the potential for ultrafast control of dynamic strain in 2D materials, on a picosecond timescale and with nanometer spatial precision, through superposition of photo-induced acoustic waves. We bridge the dynamic structural information from UEM to strain by numerical simulations using the COMSOL software package. In the simulation, we analyze the transient strain profile of a line defect. The line defect is modeled by a 3° bend of the sample, shown as the bend contour in Fig. 4(c). Following excitation, the simulations show the formation of a 20 GHz breathing mode across the entire sample, in agreement with the experimental observation (Fig. S6, supplementary material).^50^ Next, a standing wave is formed along the line defect from in-plane propagating waves. As expected from the experimental results, the center of the line defect (P1) remains stationary in the x-y plane. The P1 position corresponds to region B in the experimental analysis (node). Region P2 in the model exhibits periodic oscillations in the x-y plane, corresponding to region C in the experiments (anti-node). The spatial positions of the node and antinodes in the simulation agree well with the experimental results. Figure 4(d) shows a snapshot of the strain at 176 ps. From the simulations, we extract the volumetric strain as a function of time at the spatial positions P1 and P2. The result is traced in Fig. 4(e). An initial strain decay is observed for both P1 and P2 immediately after time zero. This is due to the application of the initial conditions of the simulation. The strain at P2 exhibits periodic oscillations, while the strain at P1 remains essentially constant. The temporal dynamics of the simulated strain are in good agreement with the structural dynamics observed in the experiments. The strain at P2 oscillates at the same frequency as the change in normalized pixel intensity of position C. When comparing the experimental results with the simulations, we observe a phase difference that begins at approximately 125 ps. This phase difference can be explained by the experimental velocity dispersion of the in-plane propagating waves, as described above, that are not included in the model. Strain modulation by photoexcitation of standing waves is not necessarily limited to line defects. A careful choice of excitation geometry, e.g., by spatially structured light,^47^ should be capable of generating standing waves throughout the sample plane. Consequently, transient strain modulation through photoinduced coherent acoustic waves could be an efficient approach for tuning the physical properties of a Weyl semimetal or other quantum materials.

## CONCLUSIONS

We determine the interplay between static strain fields, from local defects, with transient strain introduced photo-excited acoustic phonons. Through phonon superposition, the dynamic structural distortions can be modulated with a spatial distribution as fine as 20 nm. Numerical simulations, confirmed by the experimental results, describe how the local dynamic volumetric strain is tuned by the propagating acoustic wavefronts. Our experiments confirm that the strain field of Weyl semimetals can be modulated spatially, at the nanometer scale, and temporally, in the gigahertz range. Furthermore, the use of UEM allows real-time observation of structural dynamics at the picosecond scale. This work demonstrates the feasibility of manipulating transient structural properties of topological materials through light–matter interactions and simultaneous observation with UEM and paves the road for ultrafast manipulation of quantum invariants in Weyl, Dirac, and other quantum materials.

## METHOD

### Preparation of WTe_2_ sample

WTe_2_ samples were prepared by mechanical exfoliation using adhesive tape^48^ from single crystals of *Td*-WTe_2_ (2D Semiconductor, USA). Isolated flakes were transferred onto an atomically flat, cleaved (100) KCl substrate (Ted Pella). The KCl substrate was then placed in de-ionized water, leaving the flakes floating on the water surface. Subsequently, the specimens were then transferred onto a 2000-mesh Cu TEM grid (Ted Pella).

### Ultrafast time-resolved experiment

The time-resolved experiment was performed in an ultrafast electron microscope (UEM) (based on a JEOL JEM 2100) operating at 200 kV, using a hybrid pixel detector (CheeTah1800, Amsterdam Scientific Instruments). The sample was excited by a λ = 515 nm and 300 fs pulse width laser (Tangerine, Amplitude Systemes). The laser was focused to a spot with full-width at half-maximum of ∼120 *μ*m, with an average incident fluence of 2.6 mJ/cm^2^. The WTe_2_ samples were excited at a repetition rate of 20 kHz to allow for complete relaxation in-between shots. Electron probe bunches were generated through photoemission from a guard ring LaB_6_ cathode by a 258 nm laser pulse coupled to the pump pulse. Detailed information of the instrumental setup can be found in Ref. 49. The temporal width of the electron bunches was approximately 1.4 ps, as characterized by photoinduced near field electron microscopy. The time delays between the pump and probe (photoelectrons) pulses were controlled using a motorized delay stage varying the optical path length of the pump branch. The signal for each image was acquired for 100 s, or 2 × 10^6^ pump–probe cycles per frame. The experiments were performed at room temperature.

## Data Availability

The data that support the findings of this study are openly available in Zenodo at https://doi.org/10.5281/zenodo.11471263, Ref. 51.
